# Species replacement along a linear coastal habitat: phylogeography and speciation in the red alga *Mazzaella laminarioides* along the south east pacific

**DOI:** 10.1186/1471-2148-12-97

**Published:** 2012-06-25

**Authors:** Alejandro Montecinos, Bernardo R Broitman, Sylvain Faugeron, Pilar A Haye, Florence Tellier, Marie-Laure Guillemin

**Affiliations:** 1 Instituto de Ciencias Ambientales y Evolutivas, Universidad Austral de Chile, Campus Isla Teja, Casilla 567, Valdivia, Chile; 2 Departamento de Biología Marina, Facultad de Ciencias del Mar & Centro de Estudios Avanzados en Zonas Áridas (CEAZA), Universidad Católica del Norte, Larrondo 1281, Coquimbo, Chile; 3 Departamento de Ecología, Facultad de Ciencias Biológicas, Pontificia Universidad Católica de Chile, Alameda 340, Santiago, Chile; 4 Departamento de Ecología, Facultad de Ciencias, Universidad Católica de la Santísima Concepción, Casilla 297, Concepción, Chile

**Keywords:** Phylogeography, South East Pacific coast, COI, *rbc*L, Red seaweed, Parapatric distribution, Sister-species complex, Pleistocene glaciations

## Abstract

**Background:**

The Chilean shoreline, a nearly strait line of coast expanding across 35 latitudinal degrees, represents an interesting region to assess historical processes using phylogeographic analyses. Stretching along the temperate section of the East Pacific margin, the region is characterized by intense geologic activity and has experienced drastic geomorphological transformations linked to eustatic and isostatic changes during the Quaternary. In this study, we used two molecular markers to evaluate the existence of phylogeographic discontinuities and detect the genetic footprints of Pleistocene glaciations among Patagonian populations of *Mazzaella laminarioides,* a low-dispersal benthic intertidal red seaweed that inhabits along ~3,700 km of the Chilean coastal rocky shore.

**Results:**

Three main genetic lineages were found within *M. laminarioides*. They are distributed along the Chilean coast in strict parapatry. The deep divergence among lineages suggests that they could be considered putative genetic sibling species. Unexpectedly, genetic breaks were not strictly concordant with the biogeographic breaks described in the region. A Northern lineage was restricted to a broad transition zone located between 30°S and 33°S and showed signals of a recent bottleneck. The reduction of population size could be related to warm events linked to El Niño Southern Oscillation, which is known to cause massive seaweed mortality in this region. To the south, we propose that transient habitat discontinuities driven by episodic tectonic uplifting of the shoreline around the Arauco region (37°S-38°S); one of the most active forearc-basins in the South East Pacific; could be at the origin of the Central/South genetic break. The large beaches, located around 38°S, are likely to contribute to the lineages’ integrity by limiting present gene flow. Finally, the Southern lineage, occupies an area affected by ice-cover during the last glaciations. Phylogeny suggested it is a derived clade and demographic analyses showed the lineage has a typical signature of postglacial recolonization from a northern glacial refugium area.

**Conclusions:**

Even if environmental adaptation could have strengthened divergence among lineages in *M. laminarioides*, low dispersal capacity and small population size are sufficient to generate phylogeographic discontinuities determined by genetic drift alone. Interestingly, our results confirm that seaweed population connectivity over large geographic scales does not rely only on dispersal capacity but also seem to depend highly on substratum availability and population density of the receiving locality.

## Background

Relying on the assumption that genealogical breaks arose only through vicariant processes, strong phylogenetic breaks are generally associated to longstanding population separations and are thus used to pinpoint the location of (past or current) geographic barriers to dispersion. Indeed, phylogeographic discontinuities congruent across multiple taxonomic groups are often in concordance with biogeographic boundaries. Numerous studies have revealed deep genetic separation for coastal marine taxa that concur with well-known Transition Zones such as: the Isthmus of Panama [1], the south eastern Australian coast [2], the strait of Gibraltar [3], the Southern Florida Transition Zone [4] and the California Transition Zone [5,6]. These transition zones have been related to historical partitions; which are linked to eustatic, tectonic or climatic vicariant factors associated with late Pleistocene glacial cycles [4,7]. However, genetic studies constantly reveal more examples of genetic and geographic structure within taxa inhabiting continuous and homogeneous environments [8] and phylogeographic breaks have been encountered in species whose populations are continuously distributed in the absence of dispersal barriers [9,10]. In fact, regionally restricted and highly divergent phylogroups can arise at random and neutral evolutionary factors could theoretically lead ultimately to reproductive isolation and parapatric speciation without any habitat heterogeneity or geographic barrier [10-14]. Genetic drift has been implied as the main force driving local lineage sorting in species with reduced dispersal capacity and/or small population sizes. In species where geographic range size highly surpasses the mean dispersal capacity, simulations show that genetic breaks could rapidly arise and frequently involve peripheral populations [12,15]. Additionally, the existence of transient environmental discontinuities that restrict gene flow may greatly accelerate divergence processes and change the geographic location of a genetic divide [13]; explaining the frequent concordance of a phylogeographic break with biogeographic boundaries [4]. Therefore, species dispersal potential and effective population size appear to be strong determinants of the phylogeographic discontinuities magnitude; whereas any restriction to gene flow, such as biogeographic breaks, may also influence the localization of the break.

Marine intertidal species have characteristics that make them particularly susceptible to neutral processes of genetic differentiation. They have narrow and linear geographic distributions that may restrict dispersal to a stepping-stone process in species without long-lived planktonic larvae. Dispersal occurs in an advective environment (i.e. dominated by a more or less continuously oriented flow), which limits the effective population size [16]. Seaweeds have restricted dispersal capacities [17,18] while their distribution can expand from several hundred to several thousand kilometers. Indeed, recent genetic studies have indicated that genetic differentiation of seaweed populations occurs at relatively short geographic distances; ranging from <10 m to <10 km in red seaweeds [19-21] and kelps [22].

**Figure 1:**
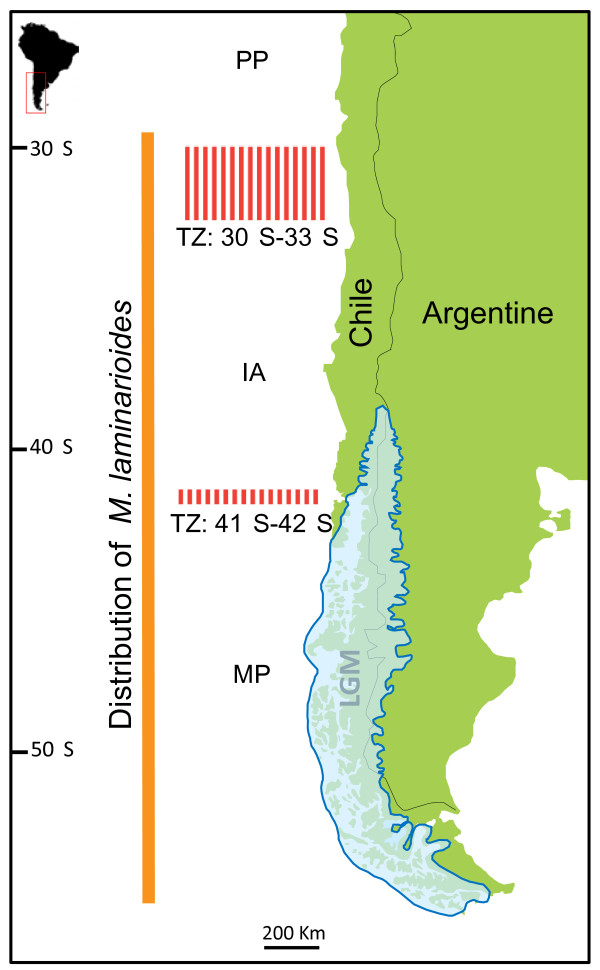
**Geographical context of the phylogeographic study on the red seaweed *Mazzaella laminarioides ***. Map of the Chilean coast showing the extension of the two biogeographic transition zones (TZ, red bars) [27]; the extent of ice during the Last Glacial Maximum (LGM; blue area) modified from [28], and the geographical distribution of M. laminarioides (orange line). Abbreviations indicate the three biogeographic provinces described for the Chilean coast: (PP) Peruvian Province, (IA) Intermediate Area, and (MP) Magellanic Province.

The South East Pacific coast (SEP) is subdivided in two major regions. North of 42°S, the coastline is linear, continuous and largely dominated by rocky shores. The coastline is also intersected by sandy beaches of up to 50 km long and a few small rivers. The oceanography and coastal climate of this region are largely defined by coastal upwelling dynamics associated with temperate east-boundary coasts [23,24]. The shoreline south of 42°S, consist of a dense array of islands, channels and fjords, strongly influenced by glacial river and sub-Antarctic oceanographic and climatic conditions. During the Quaternary period, major transformations of the coastal topography took place due to eustatic and isostatic changes [25]. An ice sheet covered an extensive region of southern Chile SEP during the last glacial-interglacial cycles, from Chiloé Island at 41°S to Cape Horn at 56°S (Figure 1) [26]. Changes in global atmospheric circulation during the Miocene-Pleistocene led to a latitudinal shift of the South Westerlies towards the Equator. These shifts were coupled with a northward advance of the sub-Antarctic biota and a range retraction of tropical/subtropical biota [23]. Camus [27] hypothesized that these events have shaped the biogeography of the Chilean coast where three main biogeographic provinces are recognized. The Peruvian Province (PP) is located on the Northern coast of Chile, from Peru to a southern limit around 30-33°S, and is dominated by a warm-temperate biota. The Magellanic Province (MP) extends from Cape Horn (56°S) up to 41-42°S (Chiloé Island), and is dominated by sub-Antarctic and cold-water species. An Intermediate Area (IA) abutted by the PP and MP provinces seems to be characterized by a diffusive overlap of biota of the two major provinces [27].

Only a handful of phylogeographic studies have been carried out on benthic species inhabiting the SEP. Contrasting genetic structure has been detected at the 41-42°S provincial boundary, such as a slight population differentiation in the giant kelp *Macrocystis pyrifera*[29] and the brooding gastropod *Acanthina monodon*[30], and a major phylogenetic break in the bull kelp *Durvillaea antarctica*[31]. Along the continuous and non-glaciated coast north of 42°S, a phylogeographic discontinuity fully concordant with the 30°S biogeographic transition was detected in the brooding gastropods *A. monodon*[30] and *Crepipatella dilatata*[32], and in the kelp *Lessonia nigrescens*[33]. In contrast, the gastropod *Concholepas concholepas,* with a long-lived pelagic larva, shows only a single and monophyletic group with a strong signal of historical demographic growth in the three biogeographic regions [34].

As observed in Northern Hemisphere benthic communities (NE Pacific, [35]; NW Atlantic, [36]; NE Atlantic, [37]), several marine species along the SEP coast have experienced local extinctions and range contractions within refuge areas located in unglaciated SEP regions at lower latitudes [29,30] and in other regions of the South Pacific Ocean (e.g. New Zealand and subAntarctic Islands: [31,38]). Species persistence in high-latitude refugia has been observed in the NE Pacific [35] and in the Northern Atlantic [37], and has also been proposed for the SEP in agreement with the potential past existence of ice-free areas near Cape Horn ([26], Figure 1). Therefore, in addition to already proposed refugia north of Chiloé Island (lower latitudes), the existence of southern refugia in the eastern part of Tierra del Fuego Island is also plausible. In the terrestrial environment, the existence of a high latitude refugia in Patagonia has been already stressed [39-42], and the genetic footprints of the Quaternary contraction/expansion have been detected in an increasing number of taxa; including plants, rodents, lizards, freshwater fishes, crabs and frogs (see [43,44] and references therein).

Here, we report on phylogeographic patterns of the intertidal red alga *Mazzaella laminarioides* (Bory) Fredericq along its entire geographic distribution ranging from 28°S to 56°S (Figure 1). Its distribution overlaps the two major biogeographic transitions of the SEP, at 42°S and 30-33°S, and extends through the formerly ice-covered area of the Last Glacial Maximum (LGM) [45]. *M. laminarioides* is a haploid-diploid rocky shore species characterized by dense beds restricted to high intertidal zones [46]. Like other intertidal organisms, *M. laminarioides* is probably highly sensitive to climatic changes and ice scour [6,38,47,48]. This species lacks floating structures and is characterized by short distance dispersal of its spores leading to small-scale genetic structure [19]. Since the presence of even a slight or transitory geographic barrier is sufficient to isolate populations in low dispersal species [43,49], we hereby predict that the gene pool of *M. laminarioides* will reflect two phylogenetic breaks linked to the ancient regional events that have originated the 30-33°S and the 42°S transition zones in the SEP. We also considered two possible scenarios of post-LGM recolonization that would lead to distinct genetic footprints: first the recolonization of the MP province from a single refugia located northern to 42°S (Chiloé Island) and second, a recolonization from two refuge areas; Chiloé Island and a remnant population located in Tierra del Fuego (56°S).

In this study we have used phylogeographic, demographic and phylogenetic inferences based on two molecular markers; the Cytochrome Oxidase I gene (COI) from the mitochondrial genome, and the large subunit of the RUBISCO gene (*rbc*L) from the chloroplast genome; to evaluate: (i) the existence of concordance between genetic and biogeographic breaks along the SEP coast in *M. laminarioides*, (ii) the effects of Pleistocene glaciations on the genetic diversity and the population demography in glaciated and un-glaciated regions, and lastly (iii) to analyze the genetic structure within the ecologically continuous habitat between 30°S and 41°S. We analyzed 352 samples from 18 populations located over the whole species’ distribution range, covering 28° of latitude (28-56°S, 3,700 km) in order to infer the historical processes that have occurred in the SEP using the peculiarity of a low-dispersal species.

## Results

### Molecular diversity and population genetic structure

For a total of 352 individuals of *M. laminarioides* sampled throughout the species’ distribution range, we detected 24 haplotypes for the mitochondrial marker COI, with 62 polymorphic sites along the 574 base pairs (bp) fragment [GenBank: JQ408408-JQ408431], (see also Additional files 1 and 2). Within the 233 individuals sequenced for the chloroplast marker *rbc*L (922 bp), we found 19 polymorphic sites and 10 haplotypes [GenBank: JQ408398-JQ408407], (see also Additional files 3 and 2).

**Figure 2:**
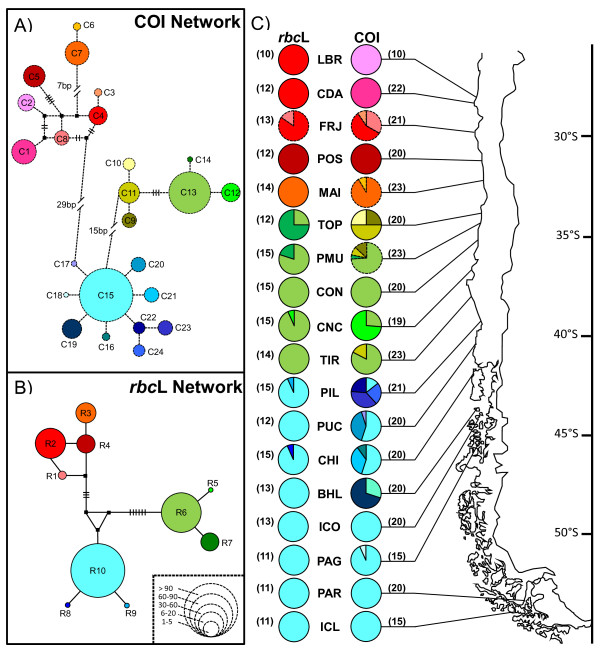
**Phylogenetic relationships among haplotypes of *M. laminarioides *and their geographic distribution based on COI and *rbc*L. ** Haplotype networks for the markers COI (**A **) and *rbc*L (**B**), and pie charts showing the geographical distribution of haplotypes (**C**). In the networks, each circle represents a haplotype and its size is proportional to the frequency in which the haplotype was encountered in each population (correspondence between circle sizes and numbers of individuals is indicated in the box B). Black squares represent hypothetical un-sampled haplotypes. For haplotypes separated by more than one mutational step, the number of steps is indicated by black bars (for values < 7), or by a number of base pairs, bp (for values ≥ 7). Pie charts’ color-code corresponds to the one used in haplotype networks. Abbreviations for population codes are as in Table 1. Numbers of sequenced individuals are given between brackets. See Additional files 1 and 3 for details.

Haplotype networks showed the presence of three main haplogroups (Figure 2A and B, for COI and *rbc*L respectively) separated by 15 to 45 bp and 6 to 10 bp for COI and *rbc*L, respectively. Within each haplogroup, pairs of haplotypes were separated by 1 to 13 bp for COI and 1 to 3 bp for *rbc*L (Figure 2A and B).

The three haplogroups were distributed parapatrically in three adjacent geographic regions: a Northern haplogroup ranging from 28°55'S to 32°37'S (5 studied localities); a Central haplogroup between 34°05'S and 37°38'S (5 studied localities); and lastly, a Southern haplogroup ranging from 39°40'S to 54°03'S (8 studied localities) (Figure 2C, see also Additional file 1 and Additional file 3). Regardless of the marker analyzed, there were no shared haplotypes among the three regions. The separation between the Northern and the Central haplogroups is located within a highly urbanized region of 212 km-long including the main ports of Valparaiso and San Antonio (~33°S). The distance separating the nearest populations of Central and Southern haplogroups is 168 km (between 37°38’S and 39°40’S). The coastal region is characterized by the presence of a sandy beach, approximately 76 km long, a habitat where *M. laminarioides* is never encountered.

For each marker, both Southern and Central haplogroups presented a single, frequent and widespread haplotype and several less frequent haplotypes. The less frequent haplotypes were predominantly restricted to a single or few nearby local populations (e.g. the *C9* and *C11* mitotypes and the *R7* chlorotype between the populations TOP and PMU, Figure 2). For the Central haplogroup, the *R6* chlorotype was detected in all surveyed localities, and the *C13* mitotype was found in all but one (the northernmost central population, TOP). For the Southern haplogroup, the *R10* chlorotype was present in all local populations analyzed, while the *C15* mitotype was detected in all but one (BHL, Figure 2). In contrast, widespread haplotypes were not found for the Northern haplogroup (Figure 2). In fact, each mitotype was restricted to a single population and the same pattern was observed for the chlorotypes in the two southernmost northern populations (i.e. MAI and POS) (Table 1, Figure 2C). The most widespread chlorotype, *R2* (Figure 2C) was detected in the three northernmost populations of the northern group, at FRJ, CDA and LBR, all spread along less than 200 km of coastline. A considerable divergence was detected between the mitotypes sequenced in the Northern haplogroup (6.96 +/− 1.68 bp) compared to what was observed within the Southern and Central haplogroups (3.13 +/− 1.16 and 2.07 +/− 0.70 bp differences between mitotypes, respectively).

**Table 1:** **Sampling sites of *M. laminarioides *and associated genetic diversity**

**Sampling site**	**Code**	**Coordinates**	**COI**						***rbc*L**					
			***N***	***nH***	***H *(SD)**	***π *(.10**^**-2**^**) (SD)**	***H***_***priv***_	***S***	***N***	***nH***	***H *(SD)**	***π *(.10**^**-2**^**) (SD)**	***H***_***priv***_	***S***
Los Burros	LBR	28°55'S/71°31'W	10	1	0	0	1	0	10	1	0	0	0	0
Chañaral	CDA	29°04'S/71°29'W	22	1	0	0	1	0	12	1	0	0	0	0
Fray Jorge	FRJ	30°40'S/71°42'W	21	3	0.581 (0.075)	0.36 (0.23)	3	5	13	2	0.282 (0.142)	0.03 (0.04)	1	1
Puerto Oscuro	POS	31°24'S/71°36'W	20	1	0	0	1	0	12	1	0	0	1	0
Maitencillo	MAI	32°37'S/71°38'W	23	2	0.166 (0.098)	0.03 (0.04)	2	1	14	1	0	0	1	0
Topocalma	TOP	34°05'S/71°58'W	20	3	0.658 (0.065)	0.14 (0.12)	1	3	12	2	0.409 (0.133)	0.09 (0.08)	0	2
Pichilemu	PMU	34°23'S/72°01'W	23	4	0.447 (0.118)	0.24 (0.17)	1	5	15	2	0.343 (0.128)	0.07 (0.07)	0	2
Constitución	CON	35°19'S/72°26'W	20	1	0	0	0	0	15	1	0	0	0	0
Concepción	CNC	36°31'S/72°57'W	19	2	0.409 (0.100)	0.07 (0.08)	1	1	15	2	0.133 (0.112)	0.01 (0.03)	1	1
Tirúa	TIR	37°38'S/73°79'W	23	2	0.300 (0.105)	0.16 (0.13)	0	3	14	1	0	0	0	0
Pilolcura	PIL	39°40'S/73°21'W	21	4	0.757 (0.048)	0.20 (0.15)	3	3	15	2	0.133 (0.112)	0.01 (0.03)	1	1
Pucatrihue	PUC	40°32'S/73°43'W	20	3	0.563 (0.063)	0.11 (0.10)	2	2	12	1	0	0	0	0
Chiloé	CHI	41°52'S/71°01'W	20	3	0.595 (0.072)	0.12 (0.10)	2	2	15	2	0.133 (0.112)	0.01 (0.03)	1	1
Bahía Low	BHL	43°47'S/73°52'W	20	2	0.442 (0.088)	0.07 (0.08)	1	1	13	1	0	0	0	0
Isla Concoto	ICO	44°11'S/73°48'W	20	1	0	0	0	0	13	1	0	0	0	0
Puerto Aguirre	PAG	45°08'S/73°30'W	15	2	0.133 (0.112)	0.02 (0.04)	1	1	11	1	0	0	0	0
Punta Arenas	PAR	53°43'S/70°57'W	20	1	0	0	0	0	11	1	0	0	0	0
Isla Clarence	ICL	54°03'S/71°58'W	15	1	0	0	0	0	11	1	0	0	0	0
**Total*M. laminarioides***			352	24	0.871 (0.012)	3.42 (0.02)	20	62	233	10	0.732 (0.019)	0.63 (0.34)	6	19
**Northern haplogroup**			96	8	0.832 (0.014)	1.07 (0.57)	8	17	61	4	0.625 (0.045)	0.01 (0.01)	4	3
**Center haplogroup**			105	6	0.619 (0.045)	0.30 (0.19)	6	7	71	3	0.308 (0.061)	0.01 (0.01)	3	3
**Southern haplogroup**			151	10	0.546 (0.047)	0.14 (0.11)	10	9	101	3	0.040 (0.030)	0.004 (0.01)	3	2

Data are indicated for each sampling site and for each haplogroup (see Figure 2). For each site, the abbreviation (**code**) and the geographic coordinates are indicated. Molecular diversity indices were calculated for the two molecular markers (COI: mitochondrial, 574 bp-long; *rbc*L: chloroplast, 922 bp-long) and are as follow: ***N***: number of sequences; ***nH***: number of haplotypes; ***H***: gene diversity; ***π***: nucleotide diversity; ***H***_***priv***_: number of private haplotypes; ***S***: number of polymorphic sites. Standard deviations (SD) are noted between brackets.

The number of mitotypes found was at least twice that of chlorotypes, regardless of the haplogroup: 8, 6 and 10 mitotypes *vs.* 4, 3 and 3 cytotypes, for the Northern, Central and Southern haplogroups, respectively (Table 1). In the same way, the estimates of molecular diversity (maximum gene diversity, *H,* and nucleotide diversity, *π*) were higher for COI than for *rbc*L, regardless of the haplogroup considered (Table 1).

Even though there was a slight tendency for a reduction of genetic diversity from the Northern haplogroup, to the Central and then to the Southern haplogroup (*H* and *π*, Table 1), these differences were not significant (Mann–Whitney *U* test, *p* > 0.05 for both the COI and the *rbc*L). Within the Southern haplogroup, a higher genetic diversity was observed in populations located in the non-glaciated area (i.e. north of 42°S: PIL, PUC and CHI) than in the five southernmost populations located within the region covered by ice during the LGM (i.e. BHL, ICO, PAG, PAR and ICL, Figures 1 and 2C, Table 1). First, for the COI data, the three northernmost populations of the Southern haplogroup exhibited a significantly higher gene and nucleotide diversity (*H* = 0.783 +/− 0.040 and *π* = 0.0023 +/− 0.0016) than the southernmost populations (*H =* 0.284 +/− 0.054 and *π* = 0.0005 +/− 0.0006) (Mann–Whitney *U* test, *p* < 0.05). Second, for *rbc*L, even if the comparison between the two regions was not significant (Mann–Whitney *U* test, *p* > 0.05), samples from local populations south of 42°S lacked genetic diversity (both *H* and *π* = 0) while three haplotypes were found in the populations north of 42°S (*H* = 0.094 +/− 0.061, *π* = 0.0001 +/− 0.0002).

Nested Analysis of Molecular Variance (AMOVA) indicated, in congruence with the results from the haplotype networks (Figure 2A, B), that total genetic variance was mainly explained by the variance among haplogroups (89.8% for COI and 94.3% for *rbc*L, Table 2); while the variance among populations within haplogroups (< 8.3%) and the variance within populations (< 1.8%), although significant, were much lower (Table 2). No significant isolation by distance was encountered, regardless of the marker and haplogroup considered (results not shown).

**Table 2:** **Analysis of molecular variance (AMOVA) of *M. laminarioides *for each molecular marker (COI and *rbc *L)**

**Source of variation**	**d.f.**	**SS**	**Variance components**	**% variation**	***p*-value**
**COI**					
Among haplogroups	2	3001.5	12.9	89.84	<0.0001
Among populations within haplogroups	15	351.3	1.2	8.33	<0.0001
Within populations	334	87.3	0.2	1.83	<0.0001
**TOTAL**	351	3440.1	14.3		
***rbc*L**					
Among haplogroups	2	626.5	4.1	94.25	<0.0001
Among populations within haplogroups	15	36.8	0.2	4.27	<0.0001
Within populations	215	13.8	0.1	1.48	<0.0001
**TOTAL**	232	677.1	4.4		

Haplogroups were defined according to the haplotype networks (see Figure 2). d.f.: degree of freedom. SS: sum of squares.

### Historical demography

**Figure 3:**
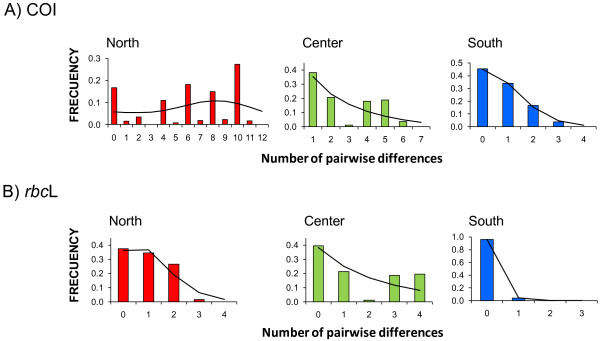
**Mismatch distributions of COI and *rbc*L data for each haplogroup of *M. laminarioides *.** Observed distributions (colored histograms) and expected distributions under a model of pure demographic expansion (black lines) of the number of pair base differences between sequences of COI (A) and *rbc*L (B) for the Northern, Center and Southern haplogroups.

**Figure 4:**
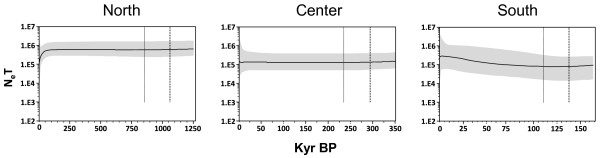
**COI Bayesian skyline plots for each haplogroup of *M. laminarioides *.** Effective population size fluctuations throughout time are represented for the Northern, Center and Southern haplogroups. The solid line represents the median estimate and the grey area the 95% highest probability density. Abbreviations used: N_e_ = effective population size, T = time, BP: before present.

For COI, a star-like network characterized the Southern haplogroup, which is usually associated with a recent population expansion (Figure 2A). The unimodal mismatch distribution fitting a population expansion model with a high bootstrap support (0.88 and 0.83 for pure demographic expansion and spatial expansion respectively, Figure 3A) and the negative values of Tajima's *D* and Fu's *F*_*s*_ statistics (*D* = −1.20, *p* = 0.100; *F*_*s*_ = −4.65, *p* = 0.003) further support a scenario of population expansion. The Bayesian skyline plot showed evidence of a subtle but persistent population growth for the Southern haplogroup (Figure 4).

The Northern and Central haplogroups appeared to have strongly distinct demographic histories when compared to the Southern haplogroup. For COI, Northern and Central haplogroups were characterized by more complex network topologies (Figure 2A), and multimodal mismatch distributions with a very low support for the two models of population expansion tested (demographic expansion and spatial expansion from 0.04 to 0.36, respectively; Figure 3A). The values obtained from the neutrality tests were positive but not significant (North: *D* = 2.42, *p* = 0.993; *F*_*s*_ = 7.76, *p* = 0.968; Center: *D* = 0.62, *p* = 0.780, *F*_*s*_ = 1.47, *p* = 0.781). The Bayesian skyline plots detected a recent population decrease in the Northern haplogroup and stable population size for the Central haplogroup (Figure 4).

The identification of a particular demographic process using *rbc*L was strongly limited by the low within haplogroup diversity. Nevertheless, results suggested a demographic expansion for the Southern haplogroup. Neutrality tests yielded negative and significant values (*D* = −1.37, *p* = 0.03; *F*_*s*_ *= −*4.04, *p* < 0.01), and the mismatch distribution was unimodal (Figure 3B), with low support for fitting the population expansion models (0.15 and 0.16 for pure demographic expansion and spatial expansion models, respectively). For the Central and Northern haplogroups, neutrality tests were not significant (North: *D =* 0.86, *p* = 0.81; *F*_*s*_ = 0.82, *p* = 0.69; Center: *D = −*0.07, *p* = 0.50; *F*_*s*_ = 0.98, *p* = 0.70) and the support values for the population expansion models were low (between 0.11 and 0.23) (Figure 3B).

### Phylogenetic relationships and estimated timing of haplogroup divergence

**Figure 5:**
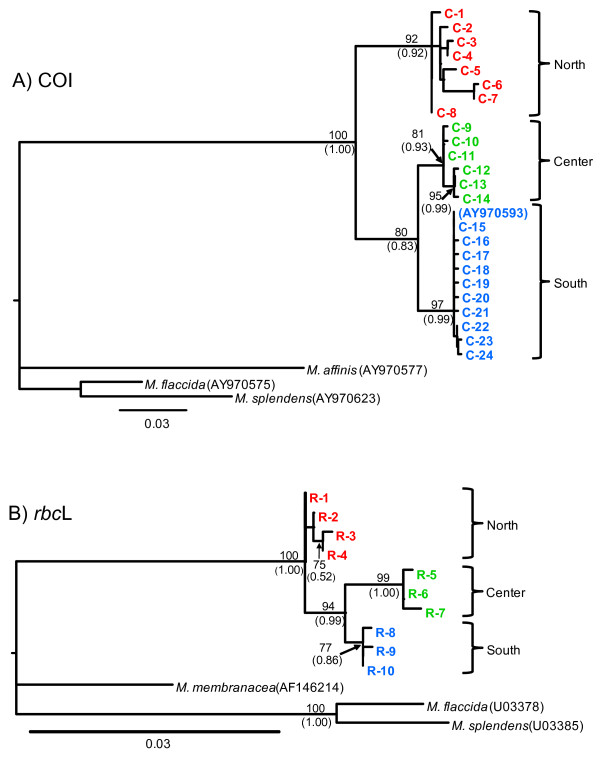
**Maximum likelihood rooted trees for COI (A) and *rbc*L (B) haplotypes of *M. laminarioides *.** Maximum likelihood bootstrap values are indicated above each node and Bayesian posterior probabilities are noted between brackets. Only high support values are shown (>75 for bootstraps and >0.75 for posterior values, respectively).

Tree topologies were broadly similar among phylogenetic reconstruction methods. For both COI and *rbc*L markers, all *M. laminarioides* haplotypes obtained in this study from 18 sampling sites formed a single well-supported monophyletic group, strongly divergent from the outgroup species (Figure 5).

In the COI tree, three monophyletic lineages were identified in *M. laminarioides*, a result fully congruent with the observation of three distinct haplogroups in the haplotype network (Figure 2A), with the Northern lineage being ancestral (Figure 5A). This complete lineage sorting (Figure 5A) and the important genetic distances separating the lineages (Kimura-two-parameters (K2P) divergence of 3.23 to 6.91%, Table 3) reflect a deep genetic divergence of haplogroups. The *rbc*L phylogeny presents two monophyletic lineages, corresponding to the Central and Southern haplogroups that together form also a monophyletic group. The Northern haplotypes formed a basal polytomy (Figure 5B).

**Table 3:** **Kimura 2-parameters (K2P) distances within- and between-haplogroups of *M. laminarioides *, for the COI and *rbc *L markers**

**Marker**	**% COI (SD)**	**%*rbc*L (SD)**
**Within-haplogroup distances**		
North	1.2312 (0.2866)	0.1812 (0.1073)
Center	0.5506 (0.2025)	0.2175 (0.1293)
South	0.3311 (0.1168)	0.1448 (0.0936)
**Mean**	0.7043 (0.2019)	0.1811 (0.1100)
**Between haplogroups distances**		
North-Center	6.9110 (1.0937)	1.3732 (0.3594)
North–South	6.5324 (1.0442)	0.7823 (0.2738)
Center-South	3.2336 (0.7111)	1.0572 (0.3052)
**Mean**	5.5590 (0.9496)	1.0709 (0.3128)
**Between *Mazzaella *recognized species**		
*Mazzaella affinis* (AY970577)	13.6298 (1.4051)	
*Mazzaella membranacea* (AF146214)		4.1582 (0.6611)

Haplogroups were defined according to the haplotype networks (see Figure 2). Standard deviations (SD) are indicated between brackets.

The GenBank sequence proposed as the COI barcode for the *M. laminarioides* species by [50] falls within the Southern lineage in the COI tree ([GenBank: AY970593], Figure 5A). This is congruent with the location where the specimen was collected, on Chiloé Island (~ 42°S). For the *rbc*L marker, two sequences from GenBank [EU082420 and EU082421] covered only a fraction of our dataset (529 bp on 922 bp). Within this fraction, only one substitution was detected between these sequences and the Northern haplotypes from our study, which is also congruent with the sampling location of these specimens (~ 32°S).

The K2P distance within each lineage ranges from 0.33 to 1.23% for the COI and from 0.15 to 0.22% for the *rbc*L (Table 3). The K2P distances between lineages were 6 and 8 times higher than those within lineages, for the COI and the *rbc*L, respectively (Table 3). The distance between lineages within the *M. laminarioides* species was however much more reduced than the one encountered between the three taxonomically recognized species of *Mazzaella* used in this study (*M. laminarioidesM. affinis,* and *M. membranacea*): 13.63 +/− 1.41% for the COI and 4.16 +/− 0.66% for the *rbc*L (Table 3). Based upon these distances, divergence dates were estimated for each monophyletic lineage found in *M. laminarioides*. For mitochondrial genes, the mutation rates varied greatly (one order of magnitude) between COI in plants and the intergenic spacer *Cox2-Cox3* in red algae [51,52]. Divergence between lineages based on COI data could be estimated as a range between 1.0 and 12.1 Myr for the Northern and Central/Southern haplogroups, and between 0.5 and 5.8 Myr for the Central and Southern haplogroups. For *rbc*L, the divergence between the Central and Southern haplogroups was dated between 8.2 and 9.6 Myr.

## Discussion

Our study revealed deep genetic structure within the red alga *Mazzaella laminarioides*, with three genetic clades distributed in strict parapatry along the Chilean coast. There was no strict concordance between the geographic distribution of lineages and two recognized biogeographic transition zones along the coast of Chile. A Southern lineage, that occupies areas that were glaciated during the LGM, showed a clear signature of postglacial recolonization from a northern refugium area.

### Three divergent haplogroups in parapatry

Despite the marker, phylogenetic trees confirmed that haplotypes of *M. laminarioides* were all grouped within a single monophyletic lineage, clearly separated from congener species. Haplotype networks of COI and *rbc*L were in complete congruence and revealed a within-species structure including three deeply divergent haplogroups with a strict parapatric distribution. The among-haplogroup distances remained largely inferior to the distance among the congener species included in our study (COI, *M. laminarioides vs. M*. *affinis:* 13.6%*; rbc*L: *M. laminarioides vs. M. membranacea*: 4.1%). However, the mean genetic distance among the three haplogroups of *M. laminarioides* is close to 7-fold of that within-haplogroups (COI: 5.50% *vs.* 0.70%; *rbc*L: 1.07% *vs.* 0.18%), confirming a deep divergence between them. The non-overlapping values of divergence of intra-lineages *vs.* inter-lineages in *M. laminarioides* are congruent with general barcode rules [53] suggesting that the lineages could be considered putative genetic sibling species. The among-lineage COI distances (from 3.2 to 6.9%) are within the range of 3.7 to 15.4% divergence reported for interspecific distances in Rhodophyta [50,54]. In the same way, a recent phylogenetic study of the genus *Mazzaella* using *rbc*L indicated an among-species divergence ranging from 1 to 6.5%, with most values around 1 to 3% of divergence [55].

Given the absence of known fossils, estimates of mutation rates are very limited and uncertain in macroalgae and our estimation of the time of divergence between *M. laminarioides* lineages should be taken with caution and as a heuristic tool. In the kelp *L. nigrescens*, a deep divergence among lineages occurring along the SEP coast has been dated to be between 0.2 and 1.7 Myr [33]. The authors suggested that this event predates the LGM. The time of divergence between *M. laminarioides* lineages, ranging from 0.5 to 12.1 Myr (depending on the marker and the divergence event considered), seems to predate Pleistocene glaciations. This is further supported by the complete lineage sorting of mitotypes and the high number of mutations accumulated between haplogroups (more than 15 for the COI). Indeed, models suggest it is unlikely that so many steps could have evolved in only 20,000 years. This is assuming a typical per gene mutation rate for the mitochondria, where a period of ≈ 50,000 years is considered a minimum for the fixation of different mitotypes in diverging populations [56]. However, small population size or directional selection lead to faster divergence rates, as has been previously reported in macroalgae [57].

Gene tree reconstructions of both COI and *rbc*L revealed some slight discordance in their topologies. For the COI, three well-supported and reciprocally monophyletic lineages were observed, while in the *rbc*L tree, the Northern haplotypes formed a set of basal parapatric branches. In fast-evolving markers (e.g. COI), the reciprocal monophyly is reached more rapidly than in slow-evolving markers (e.g. *rbc*L) and differences in mutation rates possibly explain this difference between markers [58,59]. The presence of a Southern/Central group of lineages embedded within the Northern haplotypes in the slow evolving marker *rbc*L suggests a scenario of budding speciation or parapatric speciation process [59,60] related to a southward expansion. This scenario is also supported by COI data, with the mean genetic distance among the Central and Southern haplogroups being half of the one observed between them and Northern haplogroup.

The presence of highly divergent lineages found in parapatry has been observed in other brown seaweeds such as: *Lessonia nigrescens*[33] and *Durvillaea antarctica*[31] in the SEP, *D. potatorum* on both sides of the Bassian Isthmus (Southern Australia/Tasmania littoral) [61], and *Fucus spiralis*[62] and *F. ceranoides*[63] along the North Atlantic coast of Portugal and Spain. For all these species, except *L. nigrescens*, authors have proposed that transient allopatry, as the result of geographic isolation of populations in several refugia during the Pleistocene cold cycles and subsequent recolonization, could have participated in the parapatric pattern of sister-species pair’s distribution actually observed. In the Southern hemisphere, morphological and ecological differences were noted between some of the five distinct paraphyletic clades of *D. antarctica*[64] and between East and West lineages of *D. potatorum* in Tasmania [61]. However, the presence of reproductive barriers was not tested in these species complexes. Large rates of hybridization and introgression have been noted between species of *Fucus*[62] and this genus seems to be characterized by a leaky reproductive barrier. On the other hand, there is complete reproductive isolation between the Southern and Northern lineages of *L. nigrescens*, taxa that were recently recognized as different biological species [65]. To test for reproductive isolation between the three lineages of *M. laminarioides*, new studies will be necessary, such as controlled crosses and/or estimation of potential inter-lineage gene flow considering a smaller spatial scale and hypervariable nuclear markers.

### Two phylogeographic breaks at unexpected locations

Two phylogeographic breaks were located: (1) between 32-34°S (Northern *vs.* Central haplogroups) and (2) between 37-39°S (Central *vs.* Southern haplogroups) for *M. laminarioides*, however, the biogeographic breaks along the Chilean coast are considered to be located at 30°S and 42°S.

### The northern haplogroup: Located within a “broad transition zone”?

Camus [27] (but see also [23] for a more recent review), has based the localization of the SEP biogeographic breaks on a review of published species’ range distributions of coastal invertebrates and seaweeds. Nevertheless, there is a large discordance among taxa regarding the northern break localization. Break points tend to concentrate between 30°S and 33°S [23]. It has been suggested that, the 33°S break corresponds to the range distribution limit of some red algal species. However, a biogeographic breakpoint was detected at 30°S for brown algae [66,67]. Additionally, phylogeographic discontinuities have been recently detected in several benthic species, although the genetic breaks vary in their intensity and their geographic localization: *L. nigrescens*, between 29° and 31°S [33], *Acanthina monodon*, between 30° and 33°S [30], *Notochthamalus scabrosus*, between 31° and 32°S [68], *Macrocystis pyrifera*, around 33°S [29] and *Crepipatella dilatata*, between 29° and 32°S [32]. The Northern haplogroup of *M. laminarioides*, distributed between 29°S and 34°S, seems to be characteristic of this broad ecological, biogeographical, and phylogeographical transition zone (29-33°S).

Abrupt and large changes in larval recruitment and adult abundance of key intertidal invertebrate species across the 30-33°S region have been associated with a discontinuity in near shore oceanographic and atmospheric conditions [69,70]. The region south of 30°S is characterized by strong and seasonal upwelling, while the region north of 30°S experiences less intense but more sustained upwelling [71]. Such physical changes create a strong break in circulation patterns. This is evidenced by the spatial distribution of kinetic eddy activity in the offshore [72] and near shore thermal regimes [73,74]. The ultimate spatial drivers outlined above are global circulation patterns, which control the relative positions of the South Pacific anticyclone and the southern hemisphere Westerlies [75]. The 30-33°S region, lying in the centre of these two systems, is a broad transition zone rather than a marked break.

*M. laminarioides’* Northern haplogroup presented a strong population structure and mitigated signals of demographic fluctuation. Both the skyline plots and the mismatch reconstructions indicated a recent decrease in the effective population size (although the neutrality tests were not significant). The Northern haplotypes were geographically very restricted. All mitotypes and all but one cytotypes were private. Likely, unstable populations and habitats and biotic conditions that are at the tolerance limit of *M. laminarioides*[76], explain the location of the species’ rear edge at 29°S. Field observations in the northernmost habitats occupied by *M. laminarioides* revealed a strong spatial patchiness at meso and local scales. At mesoscale, *M. laminarioides*’ populations are clearly fragmented; no individuals have been found along the 200 km of coastline separating LBR and CDA (~ 29°S) from FRJ (31°S) (pers. obs. of the authors). At local scales, within the almost continuous rocky shores of the LBR/CDA region, populations are patchy. Contrastingly, populations located south of 31°S form nearly continuous beds where rocky shores are present. The SEP coast north of 35°S is characterized by interannual cold and warm ocean temperature events linked to El Niño Southern Oscillation (ENSO), a global ocean-climate disturbance present since the Pliocene [23]. The Northern haplogroup, and particularly the northernmost populations, may have experienced repeated environmental filtering through ENSO events. These events have been shown to trigger massive seaweed mortality [77], affecting population density and genetic structure. Repeated local extinctions and range fragmentations associated with large interannual environmental variability lead to strong genetic drift. This may have accelerated the divergence process between the Northern and Central haplogroups and among the Northern haplogroup populations. However, adaptive divergence in the northern environment cannot be excluded.

### The 38°S genetic break: A genetic discontinuity of stochastic origin?

Our study revealed an ancient genetic discontinuity between the Central and Southern sister lineages. These lineages formed reciprocally monophyletic groups in both marker trees. However, between 37°S and 39°S, no biogeographic break has been described in marine organisms [23,27] and no phylogeographic break has been observed for the benthic species studied so far (seaweeds: *M. pyrifera*: [29]; *L. nigrescens*: [33]; invertebrates: *C. concholepas*[34]; *J. cirratus*[68], *A. monodon*[30]). The region bears some of the longest sections of sandy beaches along the Chilean coastline, from Peninsula Tumbes (36°47’S) to Llico (37°11’S), and from Lebu (37°36’S) to Queule (39°23’S), [23]. Only small patches of rocky shore interrupt the sand beaches within the region. This curtailed habitat availability for *M. laminarioides* is likely to contribute to haplogroup divergence. Correspondingly, large beaches have been proposed as potential barriers to gene flow for two other marine organisms, with habitat restricted to rocky intertidal shores: the southern bull-kelp *Durvillaea antarctica*[31] and the marine otter *Lontra felina*[42]. By limiting present gene flow, stretches of sandy beaches are likely to contribute to the preservation of parapatric distribution and thus, to strengthen the integrity of each lineage of *M. laminarioides*.

Uplift and subsidence are common phenomena along the temperate Eastern Pacific margin, a coastline shaped by the subduction of the oceanic Nazca plate under the South American continent. Coastal geomorphology bears evidence of the complex interplay between local tectonic uplift and glacio-eustatic sea-level changes [78]. The Arauco region (37°S-38°S), characterized by recurrent mega thrust earthquakes, has experienced rapid and constant coastal uplift since the late Pliocene and is considered as one of the most active forearc-basins along the SEP coast [79]. Rapid uplift can cause massive mortalities in the intertidal community of sessile organisms leading to repeated events of extinction and genetic bottleneck (1835: [80]; 1985: [81]; 2010: [82]). The absence of concordance between phylogenetic studies reported to date for a few marine species and this study could be due to the species-specific stochastic events caused by transient habitat discontinuity (i.e. recurrent uplifts). Habitat discontinuity may be at the origin of the Central/South genetic break in *M. laminarioides*. Along the Arauco region, the small population sizes of *M. laminarioides* (due to habitat fragmentation) together with its low dispersal capacity could have lead to phylogeographic discontinuities determined by genetic drift alone [10,13]. It is worth noting that Miocene-Pliocene tectonic processes and Andean orogeny near 38°S have been postulated as the origin of most phylogeographic patterns observed in this region for various terrestrial plants species [44].

### No phylogeographic break despite a strong biogeographic discontinuity at 42°S but the footprint of rapid post-glacial expansion

A strong biogeographic discontinuity along the Chilean coast has been described within the 41-42°S zone [27]. This discontinuity is generally related to the latitudinal migration of the southern Westerlies during the Miocene-Pleistocene. Nowadays, major ecologic, climatic and topographic discontinuities begin around this latitude. The split of the West Wind Drift into the northward Humboldt Current and the southward Cape Horn Current is a major oceanic barrier that may contribute to the maintenance of the biogeographic break. Only a handful of phylogeographic studies focusing on marine organisms have included samples from both sides of the 42°S transition zone. A phylogeographic break near this area has been described for three species: *Acanthina monodon*, brooding gastropod, [30]; *Durvillaea antarctica*, buoyant kelp, [31] and the Patagonian otter *Lontra provocax*[42]. However, no genetic divergence was detected in the buoyant kelp *Macrocystis pyrifera*[29], in *Concholepas concholepas* a gastropod with an extended pelagic larval phase [34] and in *M. laminarioides* (this study). The existence of a genetic break at 42°S seems to be less related to species dispersal capacity than to its Quaternary history in Patagonia. Indeed, for *A. monodonD. antarctica* and *L. provocax*, the 42°S biogeographic break seems to represent a secondary contact zone between haplotypes that had diverged in distinct refugia. In these species, the southernmost populations represent a recent expansion from coastal refugia located within the SEP glaciated region [30,42] or from a transoceanic source (i.e. New Zealand subantarctic region, [31]).

In *M. laminarioides*, the Southern lineage appears strongly affected by the Quaternary oscillations and shows, as expected, a star-like topology consistent with a recent demographic expansion. Populations located south of 43°S (i.e. region previously covered by ice) exhibit a very low genetic diversity. The common haplotypes present in the southernmost populations were also encountered in the Southern haplogroup populations north of 43°S. This pattern is recurrent in leading edge expansions. This type of expansion occurs when rare long-distance colonizers spread ahead of a core population, and characterizes the post LGM poleward expansion of the Northern hemisphere temperate terrestrial and marine biota [7,35,37]. We then conclude that a post-glacial recolonization from a unique refugia area, located along the SEP at the north of the Chiloé Island (from PIL to CHI, 38-41°S), has taken place in *M. laminarioides*. Ice scouring and winter freezing has a drastic impact on sessile organisms living in the high intertidal [83], and indeed *M. laminarioides* shows a complete population extirpation in Patagonia during the LGM and a severe colonization sweep in this region. The absence of a genetic break at 42°S may then be explained by the short time frame available to accumulate diversity (via new mutation) since the post LGM recolonization for Patagonian populations.

In our study, we detected sharp genetic differences between nearby populations, especially in the northern part of the species distribution, concordant with the poor dispersal capacity of *M. laminarioides*[17]. These results are congruent with previous studies evidencing low levels of gene flow at small geographic scales for *M. laminarioides*[19]. Additionally, studies of genetic structure of seaweeds indicate that genetic differentiation commonly occurs at scales of less than 10 km [22,84]. The spatial structure of genetic differentiation in the northern haplogroup of *M. laminarioides* is in direct contrast to the evidence of long-distance dispersal ability during the recolonization of the region previously covered by ice. Mature fronds of *M. laminarioides*, although not buoyant, could disperse via rafting with individuals fixed on drifting wood or fronds entangled between mats of *M. pyrifera* or *D. antarctica* (Authors’ pers. obs.).

Paradoxical dispersal capabilities have also been reported in other marine algae (*D. antarctica*, [31,83]; and *F. ceranoides*[63]. In these organisms, population connectivity over large geographic scale does not rely only on dispersal capacity (i.e. buoyancy [31]), but seems to depend more on the substratum availability and algal density of the receiving locality. The existence of available ecological space should facilitate the colonization and establishment on unoccupied rocky shores (e.g. new islands or coasts scoured by ice), while a high-density blocking effect could exclude new migrants in well-established populations [85]. Indeed, when large-scale dispersal events are strongly limited, the new lineages or haplotypes carried by the few immigrant individuals arriving in a dense population have a high probability of being quickly eliminated because of their rarity (e.g. difficulties in finding mates, elimination by genetic drift). Interestingly, in *M. laminarioides*, the existence of a blocking effect could explain the “rapid” habitat tracking of new suitable habitats during the last 17,000 years in Patagonia through rare events of large-distance dispersal by a handful of individuals on high-intertidal shores almost completely depopulated. This process may be contributing to the complete parapatric distribution of the three lineages and the lack of connectivity among established populations along the Northern part of the Chilean coast not affected by ice.

## Conclusions

Our study reveals that *M. laminarioides*, a red alga inhabiting 28° of latitude (28-56°S, 3,700 km) along the SEP coast, consisted in a complex cluster of three sibling lineages. The existence of strict parapatry suggests mechanisms limiting gene flow among close populations. A high-density blocking-effect or reproductive isolation between lineages may have helped to preserve historical phylogeographic disjunction patterns. Because of its life cycle and restricted vertical distribution in the mid-high intertidal zone, *M. laminarioides* appears highly sensitive to ENSO events and ice scour during glaciations. Both mechanisms are capable of causing massive mortality. Macroalgae endemic to the SEP coast seem to provide excellent species complexes to test hypotheses involving diversification and speciation forces. Comparative studies contrasting both sides of the Arauco region should be the focus of tests investigating geological processes as drivers of gene flow and drift.

## Methods

### Sampling

We sampled a total of 352 individuals of *M. laminarioides*, collected from 18 localities covering the whole distribution range of the species (Table 1, Figure 1). The sampling site of Los Burros (LBR, 28°55’S, Table 1) corresponds to the northernmost population identified for the species (B. Broitman, personal observation). The southernmost sampled population in Isla Clarence (ICL, 54°03’S, Table 1) stays nearby the species’ southern range edge at ~56°S [45]. Within each sampling site, we collected 10 to 23 single fronds from distinct holdfasts. In order to avoid sampling ramets we sampled holdfasts separated by at least 20 cm [19]. Tissues cut from clean healthy fronds were immediately placed into plastic bags filled with silica beds for rapid dehydration and preservation of DNA.

### DNA extraction, PCR amplification, sequencing and sequence alignment

Dried algal tissue was finely grounded in liquid nitrogen. DNA was extracted following the protocol described by [86], slightly modified by [19]. To amplify the mitochondrial Cytochrome c Oxidase I gene (COI), we initially used the primers GaZF1 and GaZR1 and PCR conditions described by [50]. Nevertheless, because of amplification difficulties for samples from the five northernmost populations, a new set of degenerated primers was designed using the PRIMER3 software [87]: amF 5’ GTTTTRGGTGGATGCATGTC 3’ and amR: 5’ TGRTAYAARATTGGRTCTCAAC 3’. The dataset used to design these new primers was constituted of two sequences obtained from the MAI population and 17 sequences from a set of populations from TOP down to PAR. While COI of individuals from populations located between TOP in the north and ICL in the south was amplified using GaZF1 and GaZR1, all individuals from LBR, CDA, FRJ, POS and MAI populations were amplified using the new amF and amR primers. For the amF/amR amplification, we used the same PCR reaction mixture as described in [50] and the following PCR conditions: an initial denaturation step at 94 °C for 5 min, followed by five cycles of 30 s at 94 °C, 30 s at 40 °C, 30 s at 72 °C, and then 25 cycles of 30 s at 94 °C, 30 s at 50 °C, 30 s at 72 °C. In total, 352 sequences of 573 bp were obtained.

Additionally, we amplified a 922 bp region of the chloroplastic gene *rbc*L, encoding the large subunit of the ribulose-1,5-bisphosphate carboxylase/oxygenase (RUBISCO) enzyme, using the primers F-rbcL and R-rbcS [88] and the PCR conditions described by [89]. Sequences were obtained for a sub-sample of 233 individuals.

For both markers, PCR amplifications were performed in a Perkin Elmer Gene Amp PCR System 9700 (Applied Biosystems, Foster City, USA). All PCR products were purified using UltraClean^TM^ DNA Purification kits (MO BIO Laboratories, Carlsbad, USA) and sequenced using the forward and the reverse amplification primers by Macrogen Inc. (Seoul, South Korea). Sequences were edited using Chromas v. 2.33 [90] and alignments were obtained using the CLUSTAL function of Mega v 5 [91].

### Genetic diversity and genetic distances

For each marker, we calculated five diversity indices for each sampled location and for each phylogenetic lineage using Arlequin v 3.5 [92]): the number of haplotypes (*h*); the number of private haplotypes (i.e. haplotypes found in a single population, *h*_priv_); the number of polymorphic sites (*S*); gene diversity (*H*, [93]) and nucleotide diversity (*π*, [94]). To test for differences in genetic diversity among regions theoretically un-covered *vs.* covered by ice during the LGM (i.e. northern *vs.* southern of 42°S), we realized a non-parametric Mann–Whitney *U* test (STATISTICA.7 software [95]).

To characterize the population genetic structure, pairwise values of *φ*_*ST*_ among locations were calculated and their significance was tested using 1000 permutations. Moreover, a nested Analysis of Molecular Variance (AMOVA, [96]) was implemented to test for the partition of genetic variance within locations, among locations within haplogroup and among haplogroups. The isolation by distance model [97] was used to test for a relationship between genetic distance (*φ*_*ST*_) and geographic distance (in km, measured along the coastline among population pairs), and its significance was tested using 1000 permutations. All these analyses were performed in Arlequin.

### Network reconstruction and historical demography

Haplotype networks were reconstructed for both genetic markers using the median-joining algorithm implemented in NETWORK v 4.510 [98]. Three complementary approaches were used to infer the historical demography of *M. laminarioides* in the entire dataset and then in each of the three haplogroups separately.

First, Tajima’s *D*[99] and Fu’s *F*_*s*_[100] statistics were calculated to detect significant past changes in population size. Significant departure from selection-drift equilibrium was tested by 1000 bootstrap replicates in Arlequin. Under the assumption of neutrality, negative values characterize populations in expansion while positive values, associated to the loss of rare haplotypes, are considered as a signature of recent bottlenecks [99,100].

Second, the observed mismatch distributions of the number of differences between pairs of sequences were compared to estimated values under a model of sudden pure demographic expansion [101] and a model of spatial expansion [102] using Arlequin. Fitting between observed and estimated mismatch distributions was calculated through a generalized least squares approach and tested by 1000 permutations. A multimodal distribution generally indicates a population in demographic equilibrium, while a unimodal distribution is associated with a recent pure demographic expansion or a range expansion.

Third, Bayesian skyline plots implemented in BEAST v 1.4.8 [103] were constructed to estimate the shape of population growth through time with 300 million of iterations. The model of nucleotide substitution used was the HKY model. Model parameters were sampled every 1000 iterations, the first 50 million iterations being discarded as burn-in. Throughout analyses, within-lineage per site mutation rate of 0.55% per Myr and a log-normal relaxed molecular clock were assumed. Demographic plots were visualized using Tracer v 1.0.1 [103].

### Phylogenetic reconstructions and estimation of divergence time

Phylogenetic reconstructions for each marker dataset were performed with the maximum likelihood (ML) method using TreeFinder [104] and a Bayesian inference (BI) using MrBayes v 3.1.2 [105]. Outgroup sequences considered were congeners of *M. laminarioides*[50,88,106]: *M. flaccida* [GenBank: AY970575 for COI and U03378 for *rbc*L], *M. splendens* [AY970623 for COI and U03385 for *rbc*L], *M. affinis* [AY970577 for COI, no data available for *rbc*L], and *M. membranacea* [AF146214 for *rbc*L, no data available for COI]. *M. membranacea*[55,106] and *M. affinis*[54] represented the closest known sister-taxa of *M. laminarioides* for *rbc*L and COI respectively. *M. flaccida* and *M. splendens* are more distantly related species from the *Mazzaella*/*Chondrus* clade [55,106]. We also compared the results obtained here with the available sequences in GenBank for our target species *M. laminarioides* [GenBank: AY970593 for COI; EU082421 and EU082420 for *rbc*L]. The two *rbc*L sequences covered only a partial fragment of our sequences alignment (529 bp on 922 bp) and were thus not included in our phylogenetic reconstructions.

ML analyses were performed using a mixed model taking into account the position of codons. For each codon position, the best-fitted substitution model was selected using the Akaike Information Criterion implemented in the ModelTest package of the TreeFinder program version 2008 [104,107]. The selected models for the *rbc*L data were TN for the first and second codon positions and J3 + I for the third codon position. For COI the selected models were J1, TN and HKY for first to third codon positions, respectively. Using TreeFinder, we performed a heuristic search in order to reconstruct two separate trees (i.e. COI and *rbc*L) and node supports were assessed by non-parametric bootstrap (1000 pseudo-replicates, [108]).

Bayesian inference was performed using the general type of the best fit model parameters defined for each dataset. Four independent analyses were run with four chains each, for ten million generations. Trees and parameters were sampled every 1000 generations and the default parameters were used to fit temperature and swapping. The first 25% of sampled trees were discarded as “burn-in” to ensure stabilization. The remaining trees were used to compute a consensus topology and posterior probability values. The split frequency (variance between the four independent runs) was below 0.0005, confirming that sampling was from the posterior probability distribution.

We estimated the Kimura-two-parameters (K2P) distance [109], using Mega5 [91] with 1000 replicates, both between- and within-haplogroups (i.e. sets of sequences grouped according to their phylogenetic affinities, see Results). Even though we are well aware that the virtual lack of fossils impede a precise calibration of the molecular clock in red algae, we used divergence rates already published for plants and red algae as an exploratory estimation of the historical events of divergence that have occurred in *M. laminarioides*. For the *rbc*L marker, a divergence rate of 0.109-0.127% per million years (Myr) has been proposed for the red alga *Caloglossa leprieurii*[110]. No divergence rates are available for the COI in Rhodophyta. We have considered the mutation rate of 0.55% per Myr proposed for the intergenic spacer *Cox2-Cox3* (a non-coding sequence neighboring the COI marker) for the red algae *Bostrychia calliptera*[51] and a divergence rate of 6.5% per Myr (mean divergence rate for core eudicots, 560 species, [52]) as lower and higher thresholds.

## Abbreviations

AMOVA: Analysis of molecular variance; BI: Bayesian inference; bp: Base pairs; COI: Cytochrome c oxidase I; df: Degree of freedom; ENSO: El Niño Southern Oscillations; IA: Intermediate Area; K2P, Kamura-2-parameters; LGM, Last Glacial Maximum; ML, Maximum Likelihood; MP: Magellanic Province; Myr: Million years; PP: Peruvian Province; rbcL: Large subunit of the RUBISCO; RUBISCO: Ribulose-1,5-bisphosphate carboxylase/oxygenase; SEP: South East Pacific; SS: Sum of squares.

## Competing interests

The authors declare that they have no competing interests.

## Authors' contributions

MLG, AM and SF designed the research. All authors contributed to field collection. AM generated sequence data and performed molecular and statistical analysis. MLG led the writing; and AM, FT and SF assisted with the writing. PH and BB revised critically the manuscript. All authors read and approved the final manuscript.

## Supplementary Material

Additional file 1 **Geographic distribution of COI haplotypes.** Number of individuals bearing each reported haplotype for COI in each sampling site (abbreviations as in Table 1). Click here for file

Additional file 2 **GenBank Accession Numbers of each COI and *rbc *L haplotype.** Click here for file

Additional file 3 **Geographic distribution of *rbc *L haplotypes. ** Number of individuals bearing each reported haplotype for *rbc *L in each sampling site (abbreviations as in Table 1). Click here for file
