# Supplementary material for: Species replacement along a linear coastal habitat: phylogeography and speciation in the red alga Mazzaella laminarioides along the south east pacific
Source: BMC Evol Biol. 2012 Jun 25;12:97. doi: 10.1186/1471-2148-12-97 (PMC3483259; doi:10.1186/1471-2148-12-97)
Supplement: Additional file 1 — Geographic distribution of COI haplotypes. Number of individuals bearing each reported haplotype for COI in each sampling site (abbreviations as in Table 1). [file 1471-2148-12-97-S1.pdf]

Additional file 1 – Geographic distribution of COI haplotypes

Number of individuals bearing each reported haplotype for COI in each sampling site (abbreviations as in Table 1).

**Haplo-group**	**Code**	**C1**	**C2**	**C3**	**C4**	**C5**	**C6**	**C7**	**C8**	**C9**	**C10**	**C11**	**C12**	**C13**	**C14**	**C15**	**C16**	**C17**	**C18**	**C19**	**C20**	**C21**	**C22**	**C23**	**C24**	**TOTAL**
Northern	LBR		10																							**10**
	CDA	22																								**22**
	FRJ			2	12				7																	**21**
	POS					20																				**20**
	MAI						2	21																		**23**
Center	TOP									5	5	10														**20**
	PMU									3		2		17	1											**23**
	CON													20												**20**
	CNC												14	5												**19**
	TIR											4		19												**23**
Southern	PIL															3							5	8	5	**21**
	PUC															11		1			8					**20**
	CHI															11	2					7				**20**
	BHL															6				14						**20**
	ICO															20										**20**
	PAG															14			1							**15**
	PAR															20										**20**
	ICL															15										**15**
**TOTAL**		**22**	**10**	**2**	**12**	**20**	**2**	**21**	**7**	**8**	**5**	**16**	**14**	**61**	**1**	**100**	**2**	**1**	**1**	**14**	**8**	**7**	**5**	**8**	**5**	**352**
