# Supplementary material for: Species replacement along a linear coastal habitat: phylogeography and speciation in the red alga Mazzaella laminarioides along the south east pacific
Source: BMC Evol Biol. 2012 Jun 25;12:97. doi: 10.1186/1471-2148-12-97 (PMC3483259; doi:10.1186/1471-2148-12-97)
Supplement: Additional file 2 — GenBank Accession Numbers of each COI and rbc L haplotype. [file 1471-2148-12-97-S2.pdf]

Additional file 3 – GenBank Accession Numbers of each COI and *rbc*L haplotype

Marker	Haplotype	GenBank Accession Number
**COI**	**C1**	JQ408408
	**C2**	JQ408412
	**C3**	JQ408411
	**C4**	JQ408410
	**C5**	JQ408413
	**C6**	JQ408415
	**C7**	JQ408414
	**C8**	JQ408409
	**C9**	JQ408417
	**C10**	JQ408421
	**C11**	JQ408418
	**C12**	JQ408420
	**C13**	JQ408416
	**C14**	JQ408419
	**C15**	JQ408422
	**C16**	JQ408430
	**C17**	JQ408423
	**C18**	JQ408428
	**C19**	JQ408431
	**C20**	JQ408427
	**C21**	JQ408429
	**C22**	JQ408424
	**C23**	JQ408425
	**C24**	JQ408426
***rbc*L**	**R1**	JQ408399
	**R2**	JQ408398
	**R3**	JQ408401
	**R4**	JQ408400
	**R5**	JQ408403
	**R6**	JQ408402
	**R7**	JQ408404
	**R8**	JQ408407
	**R9**	JQ408406
	**R10**	J1408405
