# Supplementary material for: Species replacement along a linear coastal habitat: phylogeography and speciation in the red alga Mazzaella laminarioides along the south east pacific
Source: BMC Evol Biol. 2012 Jun 25;12:97. doi: 10.1186/1471-2148-12-97 (PMC3483259; doi:10.1186/1471-2148-12-97)
Supplement: Additional file 3 — Geographic distribution of rbc L haplotypes. Number of individuals bearing each reported haplotype for rbc L in each sampling site (abbreviations as in Table 1). [file 1471-2148-12-97-S3.pdf]

Additional file 2 – Geographic distribution of *rbc*L haplotypes

Number of individuals bearing each reported haplotype for *rbc*L in each sampling site (abbreviations as in Table 1).

Haplogroup	Code	R1	R2	R3	R4	R5	R6	R7	R8	R9	R10	TOTAL
Northern	LBR		10									**10**
	CDA		12									**12**
	FRJ	2	11									**13**
	POS				12							**12**
	MAI			14								**14**
Center	TOP						3	9				**12**
	PMU						12	3				**15**
	CON						15					**15**
	CNC					1	14					**15**
	TIR						14					**14**
Southern	PIL									1	14	**15**
	PUC										12	**12**
	CHI								1		14	**15**
	BHL										13	**13**
	ICO										13	**13**
	PAG										11	**11**
	PAR										11	**11**
	ICL										11	**11**
[illegible]		[illegible]	[illegible]	[illegible]	[illegible]	[illegible]	[illegible]	[illegible]	[illegible]	[illegible]	[illegible]	[illegible]
